# Transapical aortic valve implantation in a patient with increased diameter of the aortic annulus and extensive calcification

**DOI:** 10.1186/1749-8090-8-S1-P179

**Published:** 2013-09-11

**Authors:** A Rösler, S Bertani, J Fraportti, M Sales, F Torres, M Pontes, V Lima, F Lucchese

**Affiliations:** 1 Cardiovascular Surgery, Hospital São Francisco, Porto Alegre, Brazil

## Background

TAVI is an option for patients with severe aortic stenosis and high surgical risk. The choice of prosthesis is based on anatomical measurements. We report the case of a patient who underwent TAVI with the average diameter of aortic annulus above the predefined limit.

## Methods

The patient is a 66y old male referred for evaluation of aortic stenosis, and presenting with fatigue, class III heart failure, hypertension, diabetes, 1st degree AV block, LBBB, previous stroke and moderate CKD. EuroSCORE I was 5.35% and EuroSCORE II was 1.98%. Echocardiography confirmed severe aortic stenosis, with an annulus 0.8 cm2, mean and peak systolic gradient 54 and 86 mmHg, and ejection fraction 59%. CT angiography showed a mean annulus diameter 28.4mm, sinotubular region 33.5mm, ascending aorta 41.1mm and volume of calcified region 892mm3. Given the low risk score, conventional surgery was indicated, but the patient rejected it and accepted only TAVI. Even the largest prosthesis available would require an aortic annulus diameter less than 26mm, versus 28,4mm from our patient. Despite this apparent mismatch and taking into consideration the extensive calcification and the patient's condition, the heart team, after exposing the risks to the patient and his family, indicated the transapical procedure.

## Results

The procedure was performed successfully in February 2013 by transapical access, using a Inovare prosthesis (Braile Biomédica). Heart block required implant of a dual chamber pacemaker. Echocardiography on site showed normal prosthesis function with peak gradient 21mmHg, without evidence of leak or regurgitation. In-hospital postoperative course was uneventful; the patient was discharged asymptomatic after 13 days.

## Conclusions

TAVI procedure can be performed successfully even with the increased diameter of the aortic annulus, suggesting that the presence of extensive calcifications in the valve apparatus may have an impact on the selection of the prosthesis size.

